# Urinary protein markers predict the severity of renal histological lesions in children with mesangial proliferative glomerulonephritis

**DOI:** 10.1186/1471-2369-13-29

**Published:** 2012-05-20

**Authors:** Yanhong Li, Jian Wang, Xueming Zhu, Qihua Feng, Xiaozhong Li, Xing Feng

**Affiliations:** 1 Institute of pediatric research, Children’s Hospital of Soochow University, Suzhou, China; 2 Department of nephrology, Children’s Hospital of Soochow University, Suzhou, China; 3 Department of pathology, Children’s Hospital of Soochow University, Suzhou, China; 4 Department of neonatology, Children’s Hospital of Soochow University, Suzhou, China

## Abstract

**Background:**

Several renal histopathological features, including mesangial hypercellularity, glomerulosclerosis, tubular atrophy and interstitial fibrosis, are considered to be independent predictors of end-stage renal failure in patients with glomerular diseases. Mesangial proliferative glomerulonephritis (MesPGN) is characterized by proliferations of mesangial cells with increase in mesangial matrix and/or deposits in mesangial region. The purpose of this study is to determine the association between urinary protein markers measured at the same time as renal biopsy and the severity of renal histological lesions in children with MesPGN, and to evaluate whether these markers could serve as predictors of severe renal histological lesions in this population.

**Methods:**

Ninety-eight children with MesPGN (40 with IgA nephropathy, 37 with IgM nephropathy, and 21 with MesPGN without IgA/IgM deposition) were enrolled. Urinary level of IgG, albumin, transferrin, α1-microglobulin, β2-microglobulin and *N*-acetyl-β-glucosaminidase from a morning sample before biopsy was measured.

The scores of mesangial hypercellularity, glomerulosclerosis, and tubule-interstitial damage were used to semi-quantitatively evaluate renal histological lesions.

**Results:**

The urine proteins, as independent factors associated with severe mesangial cellularity (> 5 mesangial cells/ mesangial area) were transferrin, albumin, α1-microglobulin, IgG and 24-hour total protein, with severe glomerulosclerosis (≥ 10 % glomeruli showing segmental adhesions or sclerosis) were transferrin and 24-hour total protein, and with severe tubule-interstitial damage (focal or diffuse tubular and interstitial lesions) were transferrin and *N*-acetyl-β-glucosaminidase. Urinary transferrin achieved the area under-the-receiver-operating-characteristic curve (AUC) of 0.86 and 0.82, respectively, for predicting severe mesangial cellularity and glomerulosclerosis. Urinary *N*-acetyl-β-glucosaminidase achieved the highest AUC of 0.82 for predicting severe tubule-interstitial damage. The combination of urinary protein markers, however, did not improve the predictability for renal histological lesions.

**Conclusions:**

Urinary protein markers are useful to predict the severity of renal histological lesions in children with MesPGN, which suggests that urinary proteins might be useful to predict the development and progression of renal histological lesions, and assist in evaluating the outcome and prognosis in children with MesPGN as non-invasive and easily repeatable indicators on the follow-up examination.

## Background

Mesangial proliferative glomerulonephritis (MesPGN) is characterized by proliferations of mesangial cells with increase in mesangial matrix and/or deposits in mesangial region [1,2]. IgA nephropathy (IgAN), the classic MesPGN in children, is the most common form of primary glomerulopathy and well-characterized [3-6]. IgAN is a heterogeneous disease ranging from a totally benign condition to rapidly progressive renal failure. From 15 to 40 percent of patients will eventually progress to end-stage renal disease (ESRD) [7,8]. The clinical course and pathophysiology of non-IgA MesPGN, however, has not been well described. Some investigators suggest that IgM nephropathy (IgMN) is not a distinct clinicopathologic entity, and the presence of mesangial IgM deposition is associated with a poorer treatment and worse outcome [9-11]. MesPGN without immune deposition is suggested to be a distinct type of glomerulopathy with a benign renal prognosis [12].

Accumulating studies have reported clinical, laboratory, and pathological characteristics that predict progressive renal diseases [13-16]. Histopathological features, including glomerulosclerosis, tubular atrophy, interstitial fibrosis and mesangial hypercellularity, were identified as independent predictors of ESRD in both children and adults with glomerular diseases [13,15-21]. The determination of the severity of renal histological damage in the routine follow-up in children with MesPGN may help to identify patients who will subsequently develop ESRD. Unfortunately, renal biopsy is an invasive procedure. It should not be repeatedly performed multiple times in children. Therefore, it is clinically significant to find some non-invasive indicators capable of predicting the severity of renal histological lesions in children with MesPGN.

Over the past decades, urinary protein markers, including urinary IgG, albumin, transferrin, α1-microglobulin, β2-microglobulin and *N*-acetyl-β-glucosaminidase (NAG), have been widely used in clinic to evaluate renal injury in patients with glomerular diseases. A correlation has been demonstrated between extent of various urinary proteins, renal injury and progression to renal insufficiency [14,22-24]; however, a direct demonstration of various urinary proteins predicting renal histopathological features in children is still lacking. The purpose of this study is to determine the association between urinary protein markers measured at the same time as biopsy and the severity of renal histological lesions in children with MesPGN, and to evaluate whether these markers could serve as predictors of severe renal histological lesions in this population.

## Methods

### Patients

A total of 98 children with biopsy-proven MesPGN were included in the study. All children underwent renal biopsy with automated gun under ultrasound guidance at our unit from January 2002 to December 2009. The evaluation of proteinuria was performed at the same time as biopsy. The Institutional Review Board at the Children’s Hospital affiliated to Soochow University approved the study. Written informed parental consent was obtained for each child. Exclusion criteria included secondary renal disorders (Henoch-Schönlein purpura nephritis, lupus nephritis, and haemolytic-uraemic syndrome) and biopsies with less than ten glomeruli.

Indications for renal biopsy in patients with primary glomerular diseases at our unit included persistent glomerular hematuria, asymptomatic proteinuria, steroid-dependent or -resistant nephrotic syndrome, acute nephritic syndrome presenting with non-classical features, progressive deterioration of renal function, and unexplained chronic kidney disease. The main indication for renal biopsy at our unit was asymptomatic proteinuria with hematuria, because we generally recommend renal biopsy in all these patients. The second main indication is steroid-dependent or-resistant nephrotic syndrome to identify the histopathological type. In the present study, isolated hematuria is the main clinical indication for renal biopsy (43 %), followed by asymptomatic proteinuria with or without hematuria (35 %), steroid-dependent or -resistant nephrotic syndrome (19 %) and acute nephritic syndrome (3 %).

### Clinical features

To assess the severity of clinical features, the patients were classified into five categories according to their renal manifestations at the time of biopsy: (1) micro- or macroscopic hematuria; (2) asymptomatic proteinuria with or without hematuria; (3) acute nephritic syndrome; (4) nephrotic syndrome; (5) Acute kidney injury. Classes 1 and 2 were considered as mild clinical features, and classes 3–5 as severe. Acute nephritic syndrome is defined as hematuria, red blood cell casts, proteinuria, and/or renal insufficiency, and/or hypertension. Nephrotic syndrome is defined as 24‒h proteinuria > 40 mg/m^2^/h and serum albumin level < 2.5 g/dl. Acute kidney injury is defined as a ≥ 50 % decrease in estimated creatinine clearance, based on Schwartz formula.

### Histopathological analysis

All biopsies were examined by light, immunofluorescent and electron microscopy, and reviewed and interpreted by the same pathologists. MesPGN is characterized by proliferations of mesangial cells with increase in mesangial matrix and/or deposits in mesangial region. The diagnosis of MesPGN required the presence of four or more cells per mesangial region. At least 80 % of glomeruli should be involved. The diagnosis of IgAN was based on immunofluorescent and electron microscopy sho- wing mesangial deposits of IgA as the predominant or co-dominant immunoglobulin [5,7]. IgMN was defined as primary diffuse MesPGN with IgM being deposited diffusely in the mesangium as the sole or dominant immunoglobulin on immunofluorescent and electron examination [9].

In addition to establishing the diagnosis, the following features for each biopsy were recorded: the number of total glomeruli; the total cell number per glomerulus; the number of mesangial cells; the extent of matrix expansion; the presence of endocapillary hypercellularity, inflammatory cell infiltrate, adhesion, sclerosis and crescent; the severity of tubular atrophy, interstitial inflammation and fibrosis; as well as glomerular immunofluorescent findings for IgA, IgG, IgM, complements C3 and C1q and fibrinogen graded on a scale from 0 to 3.

### Scoring of renal histological lesions

A semi-quantitative scoring for assessment of mesangial hypercellularity, glomerulosclerosis and tubule-interstitial damage (TID) was developed. Mesangial cellularity (MC) scoring, based on the number of mesangial cells on per mesangial area [15], was graded as: 1 (4–5 cells), 2 (6–7 cells) and 3 (8 or more cells). Severe MC is defined as > 5 mesangial cells/ mesangial area (MC score ≥ 2).

Glomerulosclerosis scoring was adapted from the Oxford Classification of IgA nephropathy [15,16]. Glomerulosclerosis, based on the percentage of glomeruli showing segmental adhesions or sclerosis, was scored as: 0 (absent), 1 (< 10 % of all biopsied glomeruli), 2 (10-25 % glomeruli), 3 (26-50 % glomeruli) and 4 (> 50 % glomeruli). Severe glomerulosclerosis is defined as ≥ 10 % glomeruli showing segmental adhesions or sclerosis (glomerulosclerosis score ≥ 2). “Segmental” means that a lesion involving less than half of the glomerular tuft. Adhesion is defined as an area of continuity between the glomerular tuft and Bowman’s capsule with associated extracellular matrix. Sclerosis is defined as obliteration of the capillary lumen by increased extracellular matrix [15,16,25].

TID scoring was adapted from Bazzi et al [26]. The extent of tubular atrophy, interstitial inflammation and interstitial fibrosis was scored as: 0 (absent), 1 (focal, involving < 50 % of the biopsy area), and 2 (diffuse, involving ≥ 50 % of the biopsy area), respectively. The tubular and interstitial scores were summed to obtain a single score for the TID, which was then classified as follows: 0 or 1, lesions absent or very mildly focal; 2, focal tubular and interstitial lesion; and 3 or 4, diffuse lesions. Severe TID is defined as focal or diffuse tubular and interstitial lesions (TID score ≥ 2). Tubular atrophy is defined by thick irregular tubular basement membranes with decreased diameter of tubules. Interstitial fibrosis is defined as increased extracellular matrix separating tubules in the cortical area. Interstitial inflammation is defined as inflammatory cells within the cortical interstitium in excess [15,16].

### Analysis of urinary protein markers

For each child, a first morning urine sample was obtained at the day of biopsy. Urinary level of IgG, albumin, transferrin, α1-microglobulin and β2-microglobulin were measured on an automatic biochemical analyzer (HITACHI 7600, Tokyo, Japan), and expressed in milligrams per gram of urinary creatinine (mg/g uCr). On HITACHI 7600, urinary NAG was measured using a colorimetric assay with 3-cresolsulfonphthaleyn-N-acetyl-β-D-glucosaminide as a substrate, and expressed in unit per gram of urinary Cr (u/g uCr). Urinary Cr was measured automatically by the Jaffe’s method without deproteinization. In addition, 24-hour urinary excretion of total protein (24-h proteinuria, mg/m^2^/h) was measured by Coomassie blue method.

In addition, serum level of creatinine, urea nitrogen, total protein, albumin, cholesterol, triglyceride, immunoglobulins (IgA, IgG and IgM) and complements (C3 and C4) were recorded for each child.

### Statistical analysis

Statistical analyses were performed using SPSS Statistics 13.0. Assumptions of normality and homogeneity of variance were first checked. For continuous variables with a normal distribution, descriptive results were presented as a mean and a standard deviation (SD). The t-test for unpaired samples and the one-way ANOVA were used to analyze the differences among groups. For continuous variables with a skewed distribution, descriptive results were expressed as a median and a range. The Mann–Whitney U-test and the Kruskal-Wallis test were used to analyze the difference among groups. Categorical variables were expressed as proportions. The significance of differences between proportions or percentages was determined by the Chi-square test or Fisher's exact test when the expected value was less than 5. Urinary proteins were log transformed due to a skewed distribution, when analysis of covariance (ANCOVA) was used to adjust for age, diagnosis or other covariates.

We next examined the predictive value of each urinary protein marker for severe renal histological lesions by logistic regression analysis on log-transformed data. Model fit was assessed with the Hosmer-Lemeshow goodness-of-fit test. A non-significant value for the Hosmer-Lemeshow chi-square test suggests an absence of biased fit. A receiver operating characteristic (ROC) curve was constructed and the area under the ROC curve (AUC) was calculated to assess the predictive strength. The nonparametric method of Delong was used to compare difference between AUCs [27]. Optimal cut-off points to maximize both sensitivity and specificity were also determined. The difference with *p* values < 0.05 was considered to be statistically significant. All probability values are two-sided.

## Results

### Patient characteristics

Clinical data at the time of renal biopsy and histopathological findings in 98 children with MesPGN are shown in Table 1. Of total 98 children, 40 were diagnosed with IgAN, 37 with IgMN, and 21 with MesPGN without IgA/IgM being deposited in the mesangium on immunofluorescent microscopy. Data were compared among children with IgAN, IgMN and MesPGN without IgA/IgM in Table 1. No significant difference was found in the frequency of the five categories of renal manifestations, although there was a significant difference with regard to age and body weight among groups. There was a significant difference among three groups for the frequency of mild and severe MC: 50 % of children with IgAN, 76 % with IgMN, and 76 % with MesPGN without IgA/IgM had a mild MC defined as 4–5 mesangial cells on per mesangial area (score = 1). The frequency of severe MC defined as > 5 mesangial cells/ mesangial area (score ≥ 2) was significant higher in children with IgAN.

**Table 1:** Patient characteristic at the time of renal biopsy

Characteristics		IgAN n = 40	IgMN n = 37	MesPGN without IgA/IgM n = 21	*P*
Age, years		10.2 [3.9‒15]	9.4 [3.9‒13.6]	6.2 [2.8‒13.2]	.002
Sex, male/ female		28/12	20/17	10/11	NS
Body weight, kg		33.5 [15.5‒71]	27 [14‒62.5]	20 [14‒65]	.015
Duration of disease prior to biopsy, months		2 [0.23‒120]	12 [0.33‒84]	6 [0.67‒48]	.012
Hematuria, n (%)		39 (98)	36 (97)	21 (100)	NS
Macro hematuria, n (%)		25 (63)	7 (19)	4 (19)	.000
Proteinuria > 4mg/m^2^/h, n (%)		27 (68)	18 (49)	11 (52)	NS
Proteinuria > 40mg/m^2^/h, n (%)		16 (40)	8 (22)	4 (19)	NS
Serum creatinine > 1.5mg/dl, n (%)		1 (3)	0 (0)	0 (0)	NS
Hypertension^a^, n (%)		4 (10)	2 (5)	2 (10)	NS
Clinical features, n (%)	Isolated hematuria	13 (33)	19 (51)	10(48)	NS
	Proteinuria with or without hematuria	18 (45)	10 (27)	6 (29)	NS
	Nephritic syndrome^b^	2 (5)	1 (3)	0 (0)	NS
	Nephrotic syndrome^c^	6 (15)	7 (19)	5 (24)	NS
	Acute kidney injury^d^	1 (3)	0 (0)	0 (0)	NS
Histological features, n (%)	Adhesion	12 (30)	8 (22)	8 (38)	NS
	Sclerosis	5 (13)	0 (0)	1 (5)	NS
	Crescent	1 (3)	0 (0)	0 (0)	NS
	Endocapillary hypercellularity	8 (20)	2 (5)	3 (14)	NS
	Tubular atrophy	9 (23)	5 (14)	4 (19)	NS
	Interstitial fibrosis	2 (5)	2 (5)	1 (5)	NS
	Interstitial inflammation	6 (15)	4 (11)	2 (10)	NS
	Arteriolar lesions	0 (0)	0 (0)	0 (0)	NS
	Severe MC (score ≥ 2)^e^	20 (50)	9 (24)	5 (24)	.030
	Severe GS (score ≥ 2)^f^	9 (23)	5 (14)	3 (14)	NS
	Severe TID (score ≥ 2)^g^	11 (28)	5 (14)	4 (19)	NS
	Severe MC + severe GS	6 (15)	2 (5)	2 (10)	NS
	Severe MC + severe TID	6 (15)	2 (5)	2 (10)	NS
	Severe GS + severe TID	5 (13)	3 (8)	0 (0)	NS
	Severe MC + GS + TID	3 (8)	1 (3)	0 (0)	NS
Treatmen, n (%)	Prednisone	18 (45)	14 (38)	7 (33)	NS
	Cyclophosphamide	4 (10)	3 (8)	3 (14)	NS

Values are median [range]; NS, not significant; GS, Glomerulosclerosis; MC, mesangial cellularity; TID, tubule‒interstitial damage ^a^Hypertension, defined as diastolic and/or systolic pressure higher than the 95th percentile for age, sex, and height percentile. ^b^Nephritic syndrome, defined as hematuria, red blood cell casts, proteinuria, and/or renal insufficiency, and/or hypertension. ^c^Nephrotic syndrome, defined as 24‒h proteinuria > 40mg/m^2^/h and serum albumin level < 2.5 g/dl. ^d^Acute kidney injury, defined as a ≥ 50% decrease in estimated creatinine clearance, based on Schwartz formula. ^e^Severe MC (score ≥ 2), defined as > 5 mesangial cells/ mesangial area. ^f^Severe GS (score ≥ 2), defined as ≥ 10% glomeruli showing segmental adhesions or sclerosis. ^g^Severe TID (score ≥ 2), defined as focal or diffuse tubular and interstitial lesions

### Comparison of urinary protein markers among children with IgAN, IgMN and MesPGN without IgA/IgM

In Table 2, the level of urinary albumin was significantly higher in children with IgAN as compared to those with IgMN, and those with MesPGN without IgA/IgM. The difference, however, did not remain significant after adjustment for age using ANCOVA analysis.

**Table 2:** Comparison of urinary proteins among children with IgAN, IgMN and MesPGN without IgA/IgM

Urinary protein markers	IgAN n = 40	IgMN n = 37	MesPGN without IgA/IgM n = 21	*P*
u IgG (mg/g uCr)	65.2 [2.6‒926.7]	6.5 [0.2‒397.1]	9.8 [1.6‒842.4]	NS
u Albumin (mg/g uCr)	372.8 [8–2937]	24.6 [4–1764]	53.4 [6–3300]	.021*
u Transferrin (mg/g uCr)	63.7 [0.5‒441.7]	2.5 [0.4‒804.1]	4.8 [0.3‒936.2]	NS
u α_1_‒microglobulin (mg/g uCr)	9.5 [0.1‒234.9]	5.0 [1.1‒104.5]	5.5 [0.7‒84.9]	NS
u β_2_‒microglobulin (mg/g uCr)	0.4 [0.1‒3.0]	0.4 [0.1‒1.4]	0.4 [0.0‒4.1]	NS
u NAG (u/g uCr)	17.8 [4.5‒172.0]	7.6 [2.1‒47.8]	11.8 [1.8‒48.6]	NS
24‒h Proteinuria (mg/m^2^/h)	37.0 [2.0‒257.0]	4.4 [0.5‒335.0]	37.2 [4.1‒197.3]	NS

Values are median [range]; NAG: *N*‒acetyl‒β‒glucosaminidase; NS, not significant

*The difference did not remain significant after adjustment for age using ANCOVA analysis. The level of urinary proteins was log‒transformed for ANCOVA analysis

### Comparison of clinical and laboratory parameters between children with mild and severe renal histological lesions

Of total 98 patients with MesPGN, 34 had severe MC (score ≥ 2) defined as > 5 mesangial cells/ mesangial area; 17 had severe glomerulosclerosis (score ≥ 2) defined as ≥ 10 % glomeruli showing segmental adhesions or sclerosis; and 20 had severe tubule-interstitial damage (TID) (score ≥ 2) defined as focal or diffuse tubular and interstitial lesions.

Comparison of clinical and laboratory parameters between children with mild (score < 2, n = 64) and severe MC (score ≥ 2, n = 34), between mild (score < 2, n = 81) and severe glomerulosclerosis (score ≥ 2, n = 17), and between mild (score < 2, n = 78) and severe TID (score ≥ 2, n = 20) is shown in Table 3.

**Table 3:** Comparison of clinical and laboratory parameters between children with mild and severe renal histological lesions

	Mesangial cellularity			Glomerulosclerosis			Tubule‒interstitial damage		
	Mild	Severe	*P*	Mild	Severe	*P*	Mild	Severe	*P*
Number of patients	64	34		81	17		78	20	
Age, years	9.5 [2.8‒15]	9.8 [3.9‒15]	NS	9.1 [2.8‒15]	10.4 [4.1‒14.3]	NS	8.5 [2.8‒15]	11.7 [3.1‒15]	.001
Sex (male/ female)	36/28	22/12	NS	48/33	10/7	NS	43/35	15/5	NS
Body weight, kg	30 [14‒71]	31.5 [15‒65]	NS	27.5 [14‒71	32 [15.5‒57]	NS	27 [14‒65]	37 [17‒71]	.003
	Histological diagnosis, n (%)									
IgAN	20 (31)	20 (59)	.008	31 (38)	9 (53)	NS	29 (37)	11 (55)	NS	
IgMN	28 (44)	9 (26)	NS	32 (40)	5 (29)	NS	32 (41)	5 (25)	NS	
MesPGN(IgA‒ IgM‒)	16 (25)	5 (15)	NS	18 (22)	3 (18)	NS	17 (22)	4 (20)	NS	
	Clinical features* n (%)									
Mild	55 (86)	21 (62)	.006	65 (80)	11 (65)	NS	61 (78)	15 (75)	NS	
Severe	9 (14)	13 (38)	.006	16 (20)	6 (35)	NS	17 (22)	5 (25)	NS	
	Hematuria n (%)									
5‒10RBC/HPF	11 (17)	2 (6)	NS	12 (15)	1 (6)	NS	10 (13)	3 (15)	NS	
10‒30RBC/HPF	36 (56)	23 (68)	NS	47 (58)	12 (71)	NS	43 (55)	16 (80)	NS	
> 30RBC/HPF	16 (25)	8 (24)	NS	21 (26)	3 (18)	NS	23 (29)	1 (5)	.021	
	Proteinuria, n (%)									
> 4mg/m^2^/h	29 (45)	27 (79)	.001	42 (52)	14 (82)	.021	44 (56)	12 (60)	NS	
> 40mg/m^2^/h	11 (17)	17 (50)	.001	18 (22)	10 (59)	.002	19 (24)	9 (45)	NS	
Prednisone, n (%)	18 (28)	21 (62)	.001	27 (33)	12 (71)	.004	28 (36)	11 (55)	NS	
Cyclophosphamide	4 (6)	6 (18)	NS	5 (6)	5 (29)	.013	6 (8)	4 (20)	NS	

Values are median [range]; NS, not significant.

Mild mesangial cellularity is defined as 4–5 mesangial cells on per mesangial area (score < 2); severe is defined as > 5 mesangial cells on per mesangial area (score ≥ 2).

Mild glomerulosclerosis is defined as absent or less than 10 % of all biopsied glomeruli showing segmental adhesions or sclerosis (score < 2); severe is defined as ≥ 10 % glomeruli showing segmental adhesions or sclerosis (score ≥ 2).

Mild tubule‒interstitial damage is defined as tubular and interstitial lesions absent or very mildly focal (score < 2); severe is defined as focal or diffuse tubular and interstitial lesions (score ≥ 2).

*Clinical features: the patients were classified into five categories according to their renal manifestations at the time of biopsy: (1) micro‒ or macroscopic hematuria; (2) asymptomatic proteinuria with or without hematuria; (3) acute nephritic syndrome; (4) nephrotic syndrome; (5) Acute kidney injury. Classes 1 and 2 were considered as mild clinical features, and classes 3–5 as severe.

### Comparison of urinary protein markers between children with mild and severe renal histological lesions

**Figure 1:**
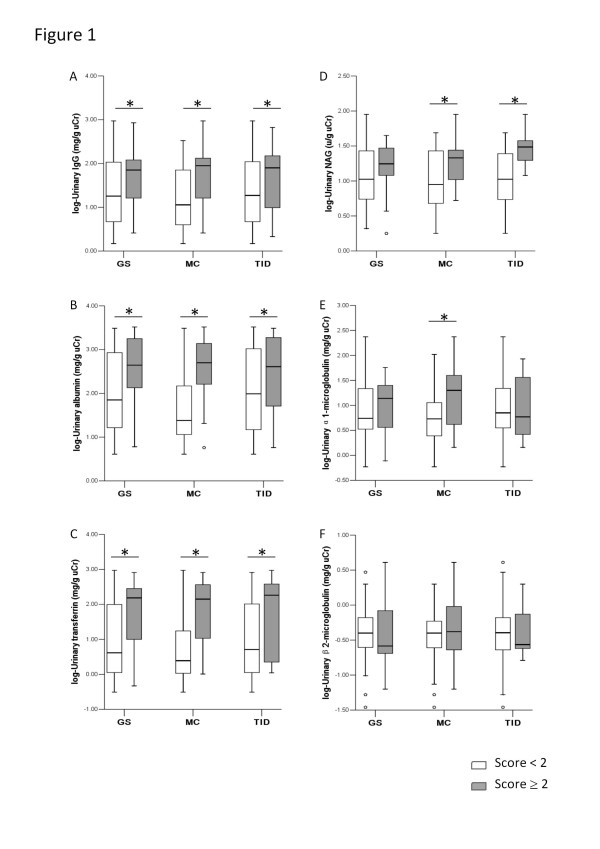
**Comparison of urinary protein markers between children with mild and severe renal histological lesions.** Comparison of urinary proteins between children with severe mesangial cellularity (MC) defined as > 5 mesangial cells/ mesangial area (score ≥ 2, n = 34) and those with mild MC (score < 2, n = 64); between children with severe glomerulosclerosis (GS) defined as ≥ 10 % glomeruli showing segmental adhesions or sclerosis (score ≥ 2, n = 17) and those with mild GS (score < 2, n = 81); and between children with severe tubule-interstitial damage (TID) defined as focal or diffuse tubular and interstitial lesions (score ≥ 2, n = 20) and those with mild TID (score < 2, n = 78). Urinary excretion of IgG (A), albumin (B), transferrin (C), NAG (D), α1-microglobulin (E) and β2-microglobulin (F) was log transformed. Values are medians, boxes represent interquartile range, whiskers indicate smallest and largest non-outlier observation, and circles indicate outliers. Probability values: two-sample Kolmogorov-Smirnov test, *p < 0.05.

As shown in Figure 1, there was a significantly higher urinary level of IgG, albumin, transferrin, α1-microglobulin and NAG in children with severe MC (score ≥ 2) when compared with children with mild MC (score < 2). The urinary level of IgG, albumin and transferrin in children with severe glomerulosclerosis (score ≥ 2) was significantly higher in comparison to children with glomerulosclerosis score < 2. The urinary level of IgG, albumin, transferrin and NAG was also significantly higher in children with severe TID (score ≥ 2), as compared to children without or with mild TID (score < 2). In Figure 1, data were log transformed.

### Association of urinary protein markers with MC, glomerulosclerosis and TID

Urinary protein markers from all patients with MesPGN (n = 98) were analyzed to predict severe MC, glomerulosclerosis and TID.

Logistic regression analysis identified that the urinary excretion of IgG, albumin, transferrin, α1-microglobulin and 24-h total protein was independently associated with severe MC defined as > 5 mesangial cells/ mesangial area (Table 4). The urinary level of β2-microglobulin and NAG was, however, not significantly associated with MC (*p* > 0.05).

**Table 4:** **Association ofurinary proteinswith renalhistological lesions**

	Odds ratio	95 % CI	P*
**Mesangial cellularity**			
u IgG (mg/g uCr)	2.86	1.36‒6.03	.006
u Albumin (mg/g uCr)	3.57	1.80‒7.07	.000
u Transferrin (mg/g uCr)	2.90	1.64‒5.12	.000
u α_1_‒microglobulin (mg/g uCr)	2.85	1.13‒7.21	.027
24‒h Proteinuria (mg/m^2^/h)	2.79	1.15‒6.81	.024
**Glomerulosclerosis**			
u Transferrin (mg/g uCr)	1.86	1.03‒3.36	.038
24‒h Proteinuria (mg/m^2^/h)	5.16	1.37‒19.43	.015
**Tubule‒interstitial damage**			
u Transferrin (mg/g uCr)	1.89	1.01‒3.54	.048
u NAG (mg/g uCr)	45.39	2.10‒980	.015

The level of urinary proteins was log‒transformed due to a skewed distribution

Odds ratio represents the increase in odds per log increase in the level of urinary proteins.

* The association remained significant after adjustment for potential confounders, including age, clinical features, histological diagnosis or treatment with prednisone and cyclophosphamide.

Urinary transferrin and 24-h proteinuria were independent factors associated with severe glomerulosclerosis defined as ≥ 10 % glomeruli showing segmental adhesions or sclerosis (Table 4). In contrast, urinary level of IgG, albumin, α1-microglobulin, β2-microglobulin and NAG was not significantly associated with glomerulosclerosis (*p* > 0.05).

Meanwhile, urinary transferrin and NAG were independently associated with severe TID defined as focal or diffuse tubular and interstitial lesions (Table 4). Urinary level of IgG, albumin, α1-microglobulin and β2-microglobulin, as well as 24-h proteinuria was, however, not significantly associated with TID in children with MesPGN (*p* > 0.05).

The odds ratio for predicting severe MC, glomerulosclerosis, and TID is shown in Table 4. The association remained significant after adjustment for age, clinical features, histological diagnosis or treatment with prednisone and cyclophosphamide as shown in Table 4.

### Ability of urinary protein markers to predict severe MC, glomerulosclerosis, and TID

In Table 5, urinary transferrin displayed the highest AUC of 0.86 (*p* = 0.000), followed by albumin, α1-microglobulin, IgG and 24-h proteinuria to predict severe MC by using ROC analysis. When combined with other urinary protein markers, the performance was not significantly better than urinary transferrin alone (the method of Delong, *p* > 0.05).

**Table 5:** Predictive characteristics of urinary proteins for severe renal histological lesions

	AUC	95% CI	*P*	Optimal cut‒off value	Sensitivity (%)	Specificity (%)
	**Severe mesangial cellularity**						
u Transferrin	0.86	0.74‒0.99	.000	45 mg/g uCr	88	74	
u Albumin	0.79	0.64‒0.95	.003	372 mg/g uCr	82	74	
u α_1_‒microglobulin	0.78	0.62‒0.94	.005	22 mg/g uCr	82	74	
u IgG	0.77	0.61‒0.92	.006	65 mg/g uCr	77	74	
24‒h Proteinuria	0.78	0.63‒0.93	.004	32 mg/m^2^/h	77	68	
	**Severe glomerulosclerosis**						
u Transferrin	0.82	0.68‒0.95	.007	136 mg/g uCr	100	74	
24‒h Proteinuria	0.79	0.64‒0.93	.014	40 mg/m^2^/h	88	61	
	**Severe tubule‒interstitial damage**						
u NAG	0.82	0.69‒0.94	.004	25 u/g uCr	75	77	
u Transferrin	0.74	0.54‒0.94	.030	97 mg/g uCr	75	77	

AUC, area under the receiver‒operating‒characteristic curve; CI, confidence interval.

Severe mesangial cellularity, defined as > 5 mesangial cells on per mesangial area.

Severe glomerulosclerosis, defined as ≥ 10 % glomeruli showing segmental adhesions or sclerosis.

Severe tubule‒interstitial damage, defined as focal or diffuse tubular and interstitial lesions.

Severe glomerulosclerosis was significantly predicted by urinary transferrin (AUC = 0.82, *p* = 0.007) and 24-h proteinuria (AUC = 0.79, *p* = 0.014). When combined with 24-h proteinuria, the performance was not significantly better than urinary transferrin alone (*p* > 0.05). Urinary NAG level was significantly predictive of severe TID (AUC = 0.82, *p* = 0.004), and better than urinary transferrin (AUC = 0.74, *p* = 0.030). When combining both markers, the performance improved (AUC = 0.92, *p* = 0.003) over that of urinary NAG alone, but not reaching statistical significance (*p* > 0.05).

### The sensitivity and specificity of urinary protein markers to predict severe MC, glomerulosclerosis, and TID based on optimal cut-off value

In Table 5, we also calculated the cut-off value for urinary proteins to predict severe MC, glomerulosclerosis and TID. Urinary transferrin displayed sensitivity 88 % and specificity 74 % at the optimal cut-off value of 45 mg/g uCr to predict severe MC. The optimal cut-off value for urinary transferrin to predict severe glomerulosclerosis was 136 mg/g uCr (sensitivity 100 %, specificity 74 %). At the optimal cut-off value of 25 u/g uCr to predict severe TID, urinary NAG displayed sensitivity 75 % and specificity 77 %.

## Discussion

In this study of children with MesPGN, we analyzed the predictive value of 6 candidate urinary proteins measured at the same time as renal biopsy, for the detection of severe renal histological lesions. Our data suggest that urinary protein markers, which have been used widely in clinic, might be useful to predict the development and progression of renal histological lesions in children with MesPGN. Mesangial cellularity, percentage of glomeruli showing segmental adhesions or sclerosis, and percentage of tubular atrophy/interstitial fibrosis were used in our study to evaluate renal histological lesions in children with MesPGN. The semi-quantitative scoring system has been utilized in previous reports [15,25,26]. According to the oxford classification of IgA nephropathy, several pathologic features could be used to interrogate prognostic significance independent of the clinical data in IgAN, which are likely to be applicable to other types of glomerulonephritis. Four of these features, including mesangial hypercellularity, glomerulosclerosis, tubular atrophy and interstitial fibrosis, were subsequently shown to have independent value in predicting renal outcome [15,16]. Furthermore, glomerulosclerosis and tubular atrophy/interstitial fibrosis are considered to be the most powerful histological predictors for the progression to ESRD in both children and adults with glomerular diseases [17-21].

Our data demonstrated that the urinary excretion of transferrin, albumin, α1- microglobulin, IgG and 24-h total protein could predict severe proliferation of mesangial cells in children with MesPGN. Urinary transferrin and 24-h proteinuria were independent factors associated with the development of glomerulosclerosis.

In our study, urinary transferrin has an increased predictive value relative to other urinary proteins as assessed by AUC, predicting the severity of mesangial cellularity and glomerulosclerosis in children with MesPGN. To our knowledge, urinary transferrin has not been studied as a marker to detect the severity of renal histological lesions. Urinary transferrin, which results from abnormal permeability of the glomerular basement membrane, is suggested to be a marker for early stages of glomerular diseases. Transferrin is very similar to albumin in molecular weight but has a higher isoelectric point. Increased urinary transferrin excretion may precede the development of microalbuminuria in glomerular diseases [28]. Although there was a significantly higher urinary level of albumin in children with severe glomerulosclerosis as compared to children with mild glomerulosclerosis, urinary albumin was not significantly associated with glomerulosclerosis by logistic regression analysis in the present study. Our result indicates that urinary transferrin may predict the severity of mesangial cellularity and glomerulosclerosis in the early stages of potentially progressive glomerular diseases.

Tubular atrophy/interstitial fibrosis are due to the increased protein traffic across the tubular cells in glomerular diseases. Accumulated evidence suggests that abnormally filtered proteins reaching the tubular lumen might be responsible for the injury of tubular cells, triggering the release of several proinflammatory cytokines, which cause interstitial inflammatory infiltration and fibrosis [14,20,29,30]. Urinary NAG, which results from the increased excretion of an isoenzyme synthesized in the tubular cells exposed to various toxic substances is useful for the detection of tubulointerstitial damage in children with MesPGN in our study. This result is in agreement with previous studies [26,31]. It has also been showed that increased NAG excretion, associated with increased transferrin excretion could occur even in the absence of morphological evidence of tubular cells damage [26,31,32].

In previous studies, the urinary excretion of α1- microglobulin, β2- microglobulin and IgG is also considered a marker for detection of tubular lesions in patients with glomerular diseases by correlation analysis [14,22,26]. Our study confirmed the correlation of various urinary proteins, except β2- microglobulin, with tubulointerstitial damage in children with MesPGN (Spearman’s correlation test, data not shown). However, by binary logistic regression analysis, only urinary excretion of NAG and transferrin, but not that of IgG, albumin, α1- microglobulin and β2- microglobulin, was significantly associated with the extent of tubulointerstitial damage.

To our knowledge, this is the first study to analyze the predictive value of urinary proteins measured at the same time as biopsy for the detection of severe renal histological lesions in children with MesPGN. Unfortunately, there were a number of limitations to our study. First, the relatively small sample size limited the power to perform logistic regression in the subgroup of IgAN or IgMN. Notably, there was no significant difference in the level of urinary proteins among the subgroups. Second, mesangial hypercellularity, glomerulosclerosis, and tubule-interstitial damage are often mixing together in actual renal biopsy findings. A total scoring system for evaluating mesangial, glomerular, tubular, and interstitial injuries would provide more prognostic information than do individual component scores. Unfortunately, the relatively small sample size limited our ability to demonstrate a significant association between urine proteins and the severity of histological lesions of MesPGN assessed by using a total score for glomerular, tubular, and interstitial injuries. Third, this study was conducted in less severely ill patients. This may raise a question whether this association is also true for a typical group of patients with severe renal manifestations. Fourth, the majority of our patients (76 %) had isolated hematuria or asymptomatic proteinuria with or without hematuria. It may not be surprising that high-molecular-weight IgG, which may reveal a severe disruption of the permeability of the glomerular capillary wall, performed less well as a predictive test in our patient population.

## Conclusions

In this study, urinary protein markers show a significant relationship with the severity of renal histological lesions. Urinary transferrin could, as an independent factor, predict severe mesangial cellularity and glomerulosclerosis. Urinary NAG could predict tubulointerstitial damage with greater accuracy than other urinary proteins in children with MesPGN. Our study suggests that on the follow-up examination, these findings have potential significance for understanding the histopathological lesions of glomeruli and tubulointerstitium in children with MesPGN without a renal biopsy. Urinary protein markers might be useful to predict the development and progression of renal histological lesions, and assist in evaluating the outcome and prognosis in children with MesPGN as non-invasive and easily repeatable indicators. Further follow-up studies are needed to confirm the association of urine protein markers and renal histological lesions with clinical outcome in children with MesPGN.

## Competing interests

The authors declare that they have no competing interests.

## Authors' contributions

L.YH had primary responsibility for study design, data analysis and interpretation and writing of the manuscript. W. J helped to draft the manuscript. Z XM participated in interpretation of renal biopsy data. F. QH participated in data collecting. L. XZ and F. X had primary responsibility for study design and protocol development. All authors read and approved the final manuscript.

## Pre-publication history

The pre-publication history for this paper can be accessed here:

http://www.biomedcentral.com/1471-2369/13/29/prepub
